# Atypical Presentation of Epidermoid Inclusion Cyst in a 60-Year-Old Female: A Case Report

**DOI:** 10.7759/cureus.29749

**Published:** 2022-09-29

**Authors:** Robert Salem, Shahzeb Ahmed, Priya Gupta, Yitong Xiao, Maryam Morris, Frederick Tiesenga

**Affiliations:** 1 Surgery, Community First Medical Center, Chicago, USA; 2 Clinical Sciences, Saint James School of Medicine, Chicago, USA; 3 General Surgery, West Suburban Medical Center, Chicago, USA

**Keywords:** asymptomatic mass, asymptomatic, thyroglossal duct cyst, neck mass, benign, epidermoid inclusion cyst

## Abstract

General surgeons frequently handle patients who present with an unknown neck mass. Due to the nature of diseases, medial neck soft tissue masses often manifest with variable etiologies and clinical signs and symptoms. Establishing a thorough evaluation of neck masses based on differential diagnoses is essential. The clinical evaluation of neck masses usually includes a thorough history and physical examination, advanced imaging techniques such as magnetic resonance imaging (MRI), ultrasonography, contrast-enhanced computed tomography (CT), and fine-needle aspiration biopsy. From a surgeon’s standpoint, proper knowledge of tissue structure, anatomy, and pathology, and applying fundamental surgical principles ensure a successful management of such lesions. This case report intends to reveal the pathological and clinical nature of an unknown neck mass in a 60-year-old female, which was postoperatively diagnosed as an epidermal inclusion cyst (EIC). This report also intends to indicate the significance of surgical intervention to prevent EIC complications and improve patient’s life quality.

## Introduction

Neck masses are often encountered in clinical practice, and the differential diagnosis of soft tissue neck masses is broad and practically evaluated based on the timeline of disease development. Based on the developmental course of the disease, the etiologies of neck masses can be categorized into acute, subacute, and chronic. By far, the diagnostic methods of neck masses are highly dependent on radiology imaging studies such as CT, which is the initial diagnostic test of choice in adults, MRI, and a fine-needle aspiration biopsy to provide diagnostic information [1]. Nevertheless, the primary goals of clinicians are to provide a thorough evaluation of every unknown neck mass encountered and to determine if the mass is malignant or benign.

Neck masses that are generally symptomatic are acute. Etiology of the acute neck mass within itself is illustrative of why it tends to be symptomatic. Hematomas are one of the causes of acute neck mass, which are created due to shearing force and trauma to the surrounding tissue and vasculature. Another etiology of acute neck mass is cervical lymphadenopathy, and, thus far, the most common cause of its lymphadenopathy is infection or inflammation [2]. Common pathologies of infectious lymphadenopathy include viruses such as Epstein-Barr Virus (EBV), Cytomegalovirus (CMV), HIV infection, and bacteria such as *Staphylococcus aureus* and group A beta *Streptococcus*. Biopsy is considered if an abnormal node has not subsided after six weeks, and should be performed promptly in patients with other associated symptoms suggestive of malignancy, such as night sweats, fever, weight loss, or a rapidly growing mass [2].

Subacute mass may propagate quickly within weeks to months. Due to the asymptomatic nature, they often go unnoticed at inception. A neck mass that is persistent and asymptomatic in an adult should be considered a malignant mass, unless proven otherwise. Due to delayed diagnosis, decreased survival rate is much more prominent in a mass such as laryngeal cancer [1]. Squamous cell carcinomas are the most common primary neoplasms of the head and neck, and their metastases is the cause of cervical lymphadenopathy. In recent years, human papillomavirus disease is becoming the predominant cause of a subset of squamous cell carcinoma. Cancers that metastasize from lungs, breasts, lymphomas, uterine cervix, gastroesophageal area, ovaries, and pancreas can cause painless cervical lymphadenopathy; however, lymphoma often progresses from being painless to painful when it rapidly grows in size. Further investigation is required if cervical lymphadenopathy is present along with risk factor, antibiotics resistance, or idiopathic etiology. Contrast-enhanced CT is the most proficient diagnostic criteria that aids in determining the stage of the lymphoma and offers possible biopsy sites [1].

Inborn masses that present in childhood can grow slowly and become prominent during adulthood [3]. Branchial cleft cysts develop anterior to the sternocleidomastoid muscle, whereas thyroglossal duct cysts (TGDCs) are midline masses situated adjacent to the hyoid bone [4]. Both these masses comprise 22% of the congenital neck masses. These masses are initially managed with antibiotics to treat any cyst infections, but definitive treatment comes in the form of surgical excision if infections occur persistently. Thyroid nodules are another cause of neck mass, but merely 5% of these are malignant and can cause chronic cervical lymphadenopathy [1]. Lipomas are soft, mobile, discrete, subcutaneous adipose tumors that may develop due to trauma usually in patients older than 35 and can rarely be found anywhere along the neck causing chronic cervical lymphadenopathy [2].

By sharing some morphological similarities with TGDC, epidermal inclusion cysts (EICs) can challenge clinicians when establishing a differential diagnosis for a patient with chronically grown neck mass such as in our case report [5]. EICs are considered as one of the most common cutaneous cysts [6]. These cysts are mobile, and they may remain stable or progressively enlarge over time. Due to its unpredictable pathological development, an EIC can enlarge, become inflamed, or remain quiescent. Once the cyst is infected, the inflammatory process will cause pain and become more noticeable to the patient [6]. In our case report, the neck mass of our 60-year-old female patient was discovered since she was 5, and the mass has not been progressing in size or other morphology changes. It was later diagnosed as EIC by pathology. Fortunately, the epidermal cyst was successfully and completely excised from our patient without any unfavored pathological development and postoperative complications. As an example in this case report, surgical removal of an EIC in the midline of the neck is considered as the most definitive treatment to prevent further clinical complications [6].

## Case presentation

A 60-year-old Hispanic female referred by her primary care provider (PCP) presented to the clinic with a chief complaint of a mass on her thyroid. The patient’s main complaint was the appearance of the mass in the midline of her neck. Although the patient had the mass since the age of 5, she denied any change in size, bleeding, discomfort, pain, erythema, or hoarseness. However, the cyst has become more visible due to the patient’s recent healthy weight loss, and the patient preferred to remove the mass for aesthetic reasons.

As per the patient, the patient immigrated to the United States many years ago from Mexico. The patient was diagnosed with diabetes mellitus type 2 recently, but denied any other medical conditions and surgeries in the past. Her family history was negative for any comorbidity, and the patient was unaware of any autoimmune diseases or cancers in her immediate family. During her recent visit with her PCP, the patient was instructed to consult with a general surgeon regarding her neck mass.

**Figure 1:**
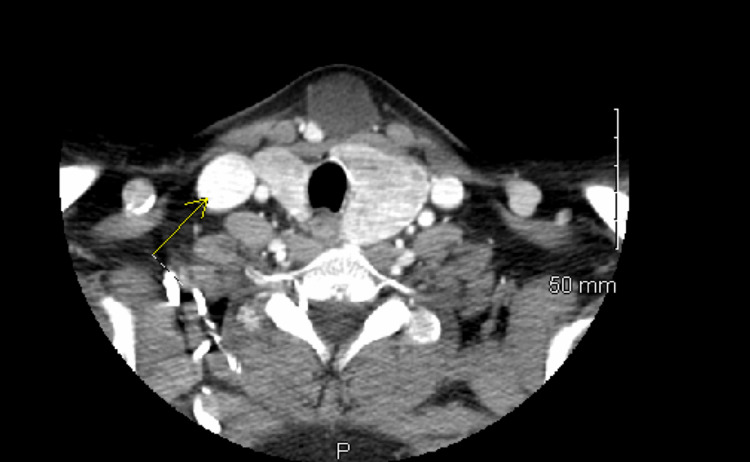
CT with contrast: a thin-walled cystic lesion was located near the thyroid gland.

**Figure 2:**
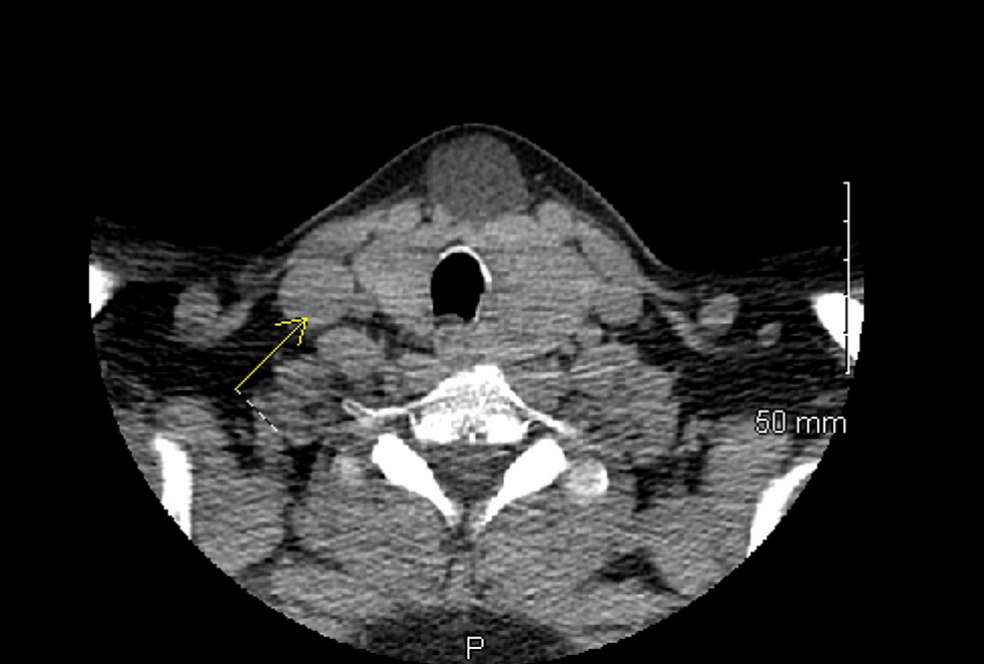
CT without contrast: a thin-walled cystic lesion was located near the thyroid gland.

A CT soft tissue neck with and without contrast using an axial scan from the skull base down to the superior mediastinum was conducted prior to surgical intervention (Figures 1, 2).

**Figure 3:**
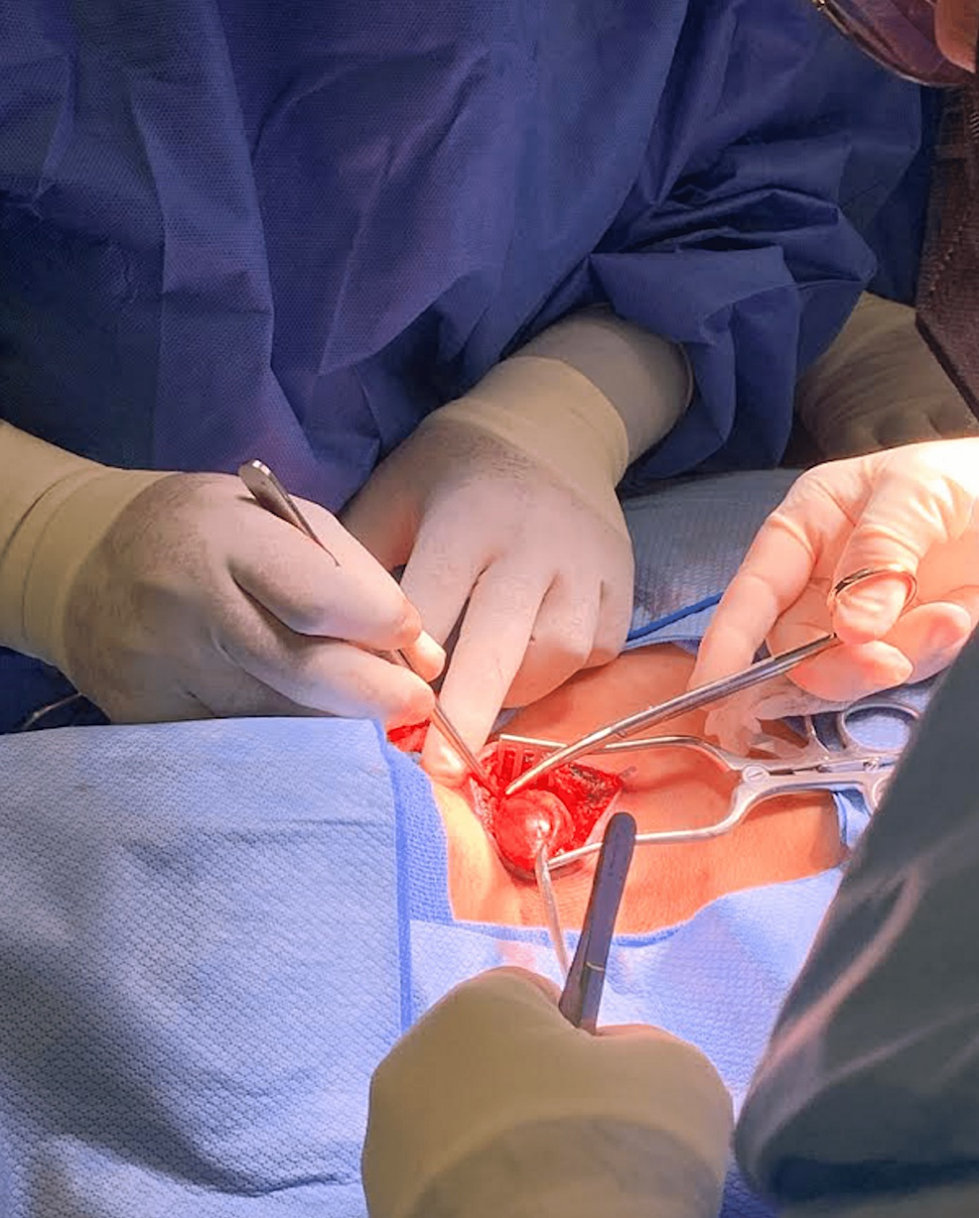
Unknown neck mass during surgical excision

Findings demonstrated a thin-walled cystic lesion at the midline of the thyroid gland just anterior to the isthmus measuring 2.2 cm x 2.8 cm (Figure 3). The lesion appears to be contiguous with the isthmus of the thyroid gland without any abnormal enhancement. There was also a large mass or goiter at the left of the thyroid measuring 3.7 cm x 3 cm x 3.5 cm. The initial impression based on the findings above included a cystic abnormality seen at midline contiguous to the isthmus of the thyroid gland. Primary considerations included a TGDC; however, a exophytic thyroid cyst from the isthmus and an epidermoid inclusion cyst could not be excluded. It was decided that the treatment plan for this patient is surgical excision of her neck mass.

The patient was taken for surgery. With her neck in extended position, a longitudinal incision was made. Hemostasis was achieved with electrocautery. The cystic mass was dissected carefully and successfully with very minimal bleeding from the surrounding tissue. The mass protruded from the hyoid bone and to the top of the trachea, and the lesion was transected flush with the hyoid bone and top of the trachea and was sent to the laboratory for further analysis. There were no complications during or after the procedure, and the patient was sent to the recovery room in stable conditions, which allowed her to be discharged later on the same day. The pathology report was received a week later with the following results: EIC (2.8 cm) with negative malignancy.

## Discussion

Although it was suspected that the neck mass we encountered was a TGDC, according to the pathology report the specimen was an EIC. While commonly found on the skin, having an EIC on the thyroid gland is a rare finding [7]. According to a study published by the National Center for Biotechnology Information (NCBI) by Palacio et al. in 2020, 85% to 90% of excised cysts are EICs [7]. However, the study also states that only 0.01% of EICs are found on mucosal sites, such as the oral cavity, and the majority are found on the trunk, neck, face, scrotum, and palmoplantar regions [7].

There are multiple similarities between EICs and TGDCs, which emphasizes the importance of thorough imaging, analysis, and treatment before making a final diagnosis. Both can present themselves as anterior neck masses [8]; however, EIC contains keratin-producing squamous epithelium, while TGDCs are predominately non-keratinizing [8]. Furthermore, the depth of the cyst is one of the most important characteristics one has to examine when considering potential differential diagnosis. This pertains specifically to our 60-year-old patient. In this case, the surgical report (mentioned above) showed the neck mass protruded to the hyoid bone and to the top of the trachea, which is an uncommon finding for EICs as it has been shown in previous literature. EICs are normally localized to the dermis or subcutaneous tissues [8], and it is more common for TGDCs to be situated in the deeper soft tissues protruding in and around the hyoid bone [8].

Another differential diagnosis closely related to EICs are dermoid cysts, which were also considered in our differential diagnosis for this patient. Both epidermal and dermoid cysts arise from squamous epithelial lining [9]. Both types of cysts arise from the dermal elements of the first and second branchial arches, which explains their location being at the base of the tongue and superficially confined to the subcutaneous tissues (in this case the anterior of the neck) [9]. However, one of the main distinguishing factors is that dermoid cysts tend to include a high fat and calcific content internally, while EICs maintain mostly a diffusion restriction [9].

As a clinician, one of the most important aspects of diagnosis of a thyroid mass is to rule out malignancy. We previously managed to differentiate between EIC and other types of cysts, and now we will shift to thyroid cancer to conclude our discussion. The patient first noticed the mass in early childhood, around the age of 5, and believes that the mass has not changed in appearance and size and has not caused any pain during the years. Based on this information, the suspicion of cancer in our 60-year-old Hispanic patient was very low, and the postoperative pathology confirmed its benign characteristics.

The JAMA Internal Medicine journal issued a retrospective case-control study from January 2000 through March 2005 discussing the results of 8,806 patients who underwent 11,618 thyroid ultrasounds. The importance of this study not only highlights the incidence of thyroid nodules and thyroid cancer but also emphasizes the importance of the variation in management starting from the patient’s first visit to the final diagnosis and treatment. Thyroid nodules were present in approximately 96.9% of patients who had cancer [10]. Out of the 11,618 ultrasounds performed, there were three characteristics that were apparent in patients who had thyroid cancer, which included microcalcifications, mass greater than 2 cm, and solid composition [10]. All other nodule characteristics were found to be irrelevant and unrelated to the patients having thyroid cancer. Lastly, studies have shown that thyroid cancer has a very promising prognosis with a survival rate of 97% [10].

## Conclusions

Epidermoid inclusion cyst on the thyroid gland is a rare finding. Nevertheless, it should be kept in mind as a possible diagnosis. History of the presenting mass plays an important role in guiding us to the right diagnosis. Although imaging modalities are necessary for diagnosis, they are not always able to differentiate between multiple differential diagnoses, such as in our case, where imaging was not able to differentiate between thyroglossal cyst, exophytic thyroid cyst, and epidermoid cyst.
